# Antagonistic, overlapping and distinct responses to biotic stress in rice (*Oryza sativa*) and interactions with abiotic stress

**DOI:** 10.1186/1471-2164-14-93

**Published:** 2013-02-12

**Authors:** Reena Narsai, Chuang Wang, Jie Chen, Jianli Wu, Huixia Shou, James Whelan

**Affiliations:** 1 Centre for Computational Systems Biology, Bayliss Building M316 University of Western Australia, 35 Stirling Highway, Crawley 6009, Western Australia, Australia; 2 ARC Centre of Excellence in Plant Energy Biology, Bayliss Building M316 University of Western Australia, 35 Stirling Highway, Crawley 6009, Western Australia, Australia; 3 Joint Research Laboratory in Genomics and Nutriomics, Zhejiang University, Hangzhou 310058, China; 4 College of Life Sciences, Zhejiang University, Hangzhou 310058, China; 5 China National Rice Research Institute, Hangzhou 310006, China; 6 ARC Centre of Excellence in Plant Energy Biology, Centre for Computational Systems Biology, MCS Building M316 University of Western Australia, 35 Stirling Highway, Crawley 6009, Western Australia, Australia

## Abstract

**Background:**

Every year, substantial crop loss occurs globally, as a result of bacterial, fungal, parasite and viral infections in rice. Here, we present an in-depth investigation of the transcriptomic response to infection with the destructive bacterial pathogen *Xanthomonas oryzae* pv. *oryzae(Xoo)* in both resistant and susceptible varieties of *Oryza sativa*. A comparative analysis to fungal, parasite and viral infection in rice is also presented.

**Results:**

Within 24 h of *Xoo* inoculation, significant reduction of cell wall components and induction of several signalling components, membrane bound receptor kinases and specific WRKY and NAC transcription factors was prominent, providing a framework for how the presence of this pathogen was signalled and response mounted. Extensive comparative analyses of various other pathogen responses, including in response to infection with another bacterium (*Xoc)*, resistant and susceptible parasite infection, fungal, and viral infections, led to a proposed model for the rice biotic stress response. In this way, a conserved induction of calcium signalling functions, and specific WRKY and NAC transcription factors, was identified in response to all biotic stresses. Comparison of these responses to abiotic stress (cold, drought, salt, heat), enabled the identification of unique genes responsive only to bacterial infection, 240 genes responsive to both abiotic and biotic stress, and 135 genes responsive to biotic, but not abiotic stresses. Functional significance of a number of these genes, using genetic inactivation or over-expression, has revealed significant stress-associated phenotypes. While only a few antagonistic responses were observed between biotic and abiotic stresses, e.g. for a number of endochitinases and kinase encoding genes, some of these may be crucial in explaining greater pathogen infection and damage under abiotic stresses.

**Conclusions:**

The analyses presented here provides a global view of the responses to multiple stresses, further validates known resistance-associated genes, and highlights new potential target genes, some lineage specific to rice, that play important roles in response to stress, providing a roadmap to develop varieties of rice that are more resistant to multiple biotic and abiotic stresses, as encountered in nature.

## Background

Every year, potential crop yields are lost as a result of exposure to devastating conditions from extreme temperature to bacterial pathogens [1,2]. Given the ever-increasing demand for food, prevention of losses from abiotic and biotic stresses offers a resource neutral avenue in terms of resource input to increasing food production. Rice is a cereal crop species that is a significant part of the staple diet for half of the world’s population and is grown on every continent apart from Antarctica [3]. Considering this vast area of growth, rice is constantly exposed to interaction with various organisms from insects to bacteria. The ability to maintain or increase rice production in a cost effect manner will rely on developing varieties that can be productive in response to a variety of abiotic or biotic stresses. The use of biotechnological approaches to develop crops resistant to a given stress imposition often takes a single gene approach [1,4], where stress induced genes encoding proteins, often transcription factors, are over-expressed in transgenic plants, resulting in greater tolerance to a given stress imposition. While this is promising there are several barriers translating laboratory based experiments to field situations, including the use of model plants compared to crop plants and the impositions of single stresses compared to multiple stresses [1].

As rice is both a model and crop plant, it offers a system to directly determine the effect of stress on growth and yield in specific field varieties, even if under laboratory conditions. Bacterial leaf blight is a common problem seen in rice species infected with *Xanthomonas oryzae* pv. *oryzae* (*Xoo*) and these infections are known to result in significant crop loss, ranging up to 60% of potential yield, or several billions in direct economic terms [2]. Given that different cultivars of rice have been observed as having different levels of resistance to infections, understanding how this is possible and the mechanisms behind resistance is important for the prevention of this problem, and provides a good reference point to determine overlap in responses to other biotic and abiotic stresses. A significant number of studies examining bacterial infection in rice has led to the identification of more than 30 resistance (R) genes in rice, which are largely annotated by the prefix *Xa*, [5-7]. Given that plant immunity is based on the recognition and constant surveillance of pathogens through the pathogen recognition receptors (PRRs) and nucleotide-binding site leucine-rich repeat (NB-LRR) type proteins (involved in pathogen effector recognition), the R genes have been observed to largely encode these defence signalling functions [5-7]. However, in addition to recognition and signalling functions being crucial to resistance, a number of WRKY transcription factors have also been observed to result in greater resistance when over-expressed/knocked-out, indicating that these have an important role in the regulation of gene expression following pathogen infection [8-10]. One example of this is for WRKY13, which has been found to be an important regulator of rice interaction with *Xoo* as well as the fungus, *M.grisea*, where activation of this gene resulted in increased resistance of rice to these infections [11]. Given the crucial role of transcription factors that have been shown to have a direct effect on resistance, as well as the findings that transcriptomic changes are characteristic of responses to infection [12-15], the examination of global transcriptomic responses provide great insight into the mode of response to infection.

Recent studies have examined the significant transcriptomic responses seen after rice infection with parasites (e.g. *Striga hermonthica*; [14]), fungus (e.g. *Magnoporithea grisea*; [13]), virus (e.g. Rice Stripe Virus (RSV); [12]) and bacteria (*Xanthomonas oryzae* pv. *oryzicola* (Xoc); [16]). In each of these studies, significant changes were seen to occur in the rice transcriptome in response to infection, with a number of genes encoding pathogenesis-related functions seen to be differentially expressed [13,14]. Following parasite infection, significant differences in the transcriptome were observed between two different cultivars of rice, one of which is known to be resistant to *S. hermonthica* (cv. Nipponbare) and one known to be susceptible (IAC65) [14]. Thus, while there are a number of studies examining the response of rice to individual biotic and abiotic stresses, this is little or no analyses of the comparative nature of the responses in terms of common, distinct or antagonistic in nature.

In this study the transcriptomic response to infection with the bacterium *Xanthomonas oryzae* pv. *oryzae (Xoo)* provides insight into the immediate changes that occur following infection, revealing that specific cell wall functions show a rapid down-regulation in response to infection in the resistant cultivar, while translation and stress-related functions were up-regulated. Furthermore, the collation and comparison of the expression responses across various other abiotic stress transcriptomic studies (Cold, drought, salt - [17]; Heat – [18]) and biotic stress transcriptomic studies, enabled the identification unique gene-sets that are responsive to bacterial infection only (this study) and a conserved response to all biotic stresses only (Bacteria, Xoo – this study; Xoc - [16]; parasite, *S.hermonthica* - [14]; virus – GSE11025; fungus, *M.grisae* - [13], *M.oyzae* – [19]). In this study we examine global transcriptomic responses to a variety of biotic (and abiotic) stresses in parallel, revealing the specific pathways e.g. calcium signalling and WRKY and NAC transcription factors that have a conserved response across multiple pathogen infections in rice. Together, these analyses enabled a model for the rice biotic stress response to be generated, showing all the pathways that are conserved in response to combinations of different pathogen infections, revealing the core pathogen response, which could not have been identified without these multiple comparisons. The functional role for several genes identified by the analyses in this study e.g. the specific WRKY and NAC transcription factors, calcium signalling proteins and metal transporters have, in recent years, been shown to have a functional role in the relevant biotic (and/or abiotic) stress response, supporting their identification by the analyses in this study, and their crucial role in the plant defence response [10,20-22]. Thus, the results presented here are not only validated by a number of these studies, that have altered the expression of single genes and observed resistance to biotic stresses [10,20-22], but also presents novel candidate genes that may also function in multiple biotic stress resistance.

## Results

### Confirming infection by *Xanthomonas oryzae* pv*. oryzae (Xoo)*

**Figure 1:**
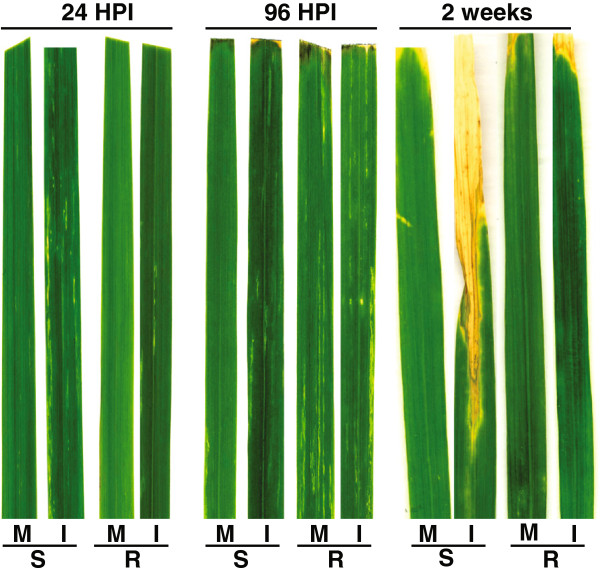
**Rice leaves were infected with bacteria (*X*. *oryzae *pv. *oryzae *- PXO71) 35 days after sowing.** Photographs revealing physical differences in appearance after infection (hours post infection). I=infected, M=mock (not infected), S=Susceptible; IR24, R=Resistant; IRBB21.

It has been shown that the rice cultivar IR24 is susceptible to infection with almost all *Xoo* strains [23,24], where infection is seen to result in significant leaf damage. In contrast, the isogenic line IRBB21 is known to be resistant to *Xoo* infection as it carries the resistance gene *Xa21*[25]. To gain insight into the early transcriptomic responses to *Xoo* infection in these two contrasting cultivars, leaves were sampled at 24 and 96 hours post infection (HPI). It is evidenced however, that although transcriptomic responses were occurring at these times, no visible differences in leaves were seen both at 24 and 96 HPI (Figure 1). However, continued examination of the infected leaves reveals that by 2 weeks after infection, significant changes in colour and cellular morphology are evidenced (Figure 1). Specifically, it can be seen that in the susceptible IR24 cultivar, greater than half of the leaf has lost colour and viability, in contrast to the mock treated IR24 and resistant IRBB21 cultivar (Figure 1). Notably, although some disease symptoms were seen in the resistant cultivar (R), it was considerably less than the damage seen in the susceptible (S) cultivar (Figure 1). Therefore, the transcriptomic responses at 24 and 96 HPI represents the earliest responses to infection, occurring at the molecular level, well before any significant morphological changes can be observed.

### Differential response to *Xoo* infection in resistant and susceptible rice cultivars

**Figure 2:**
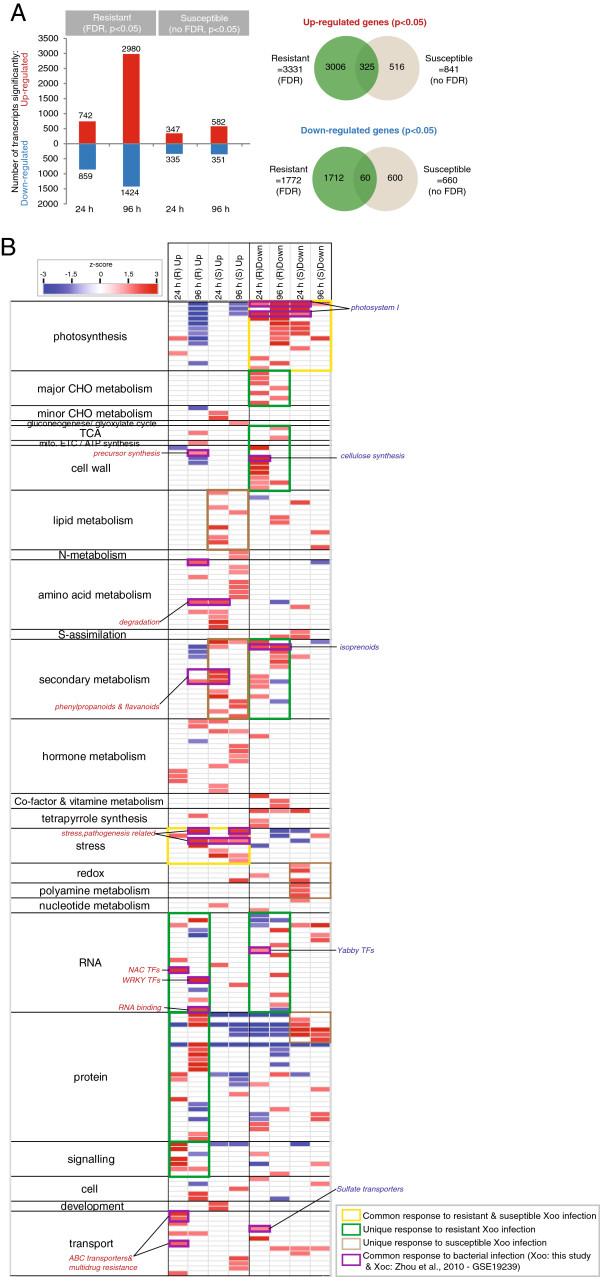
**Differential expression of transcripts after infection. A**) Number of significantly differentially expressed genes (p<0.05; PPDE>0.96). Note: PPDE threshold was not passed for the susceptible cultivar comparisons. **B**) Pageman analysis showing over/under represented functional categories, for the sets of transcripts differentially expressed after infection. Fisher’s exact test was used to determine over-represented functional categories and scores are displayed as a heatmap, where a score of 1.96 represents a p-value of 0.05.

Analysis of the transcriptomic response to infection with *Xoo* revealed that 3331 genes were significantly (p<0.05; PPDE>0.96) up-regulated and 1772 genes were down-regulated in the resistant cultivar, whilst susceptible infection resulted in less than a quarter of that number (Figure 2A; Additional file 1: Table S1). It has been suggested that susceptible response to infection is comparable to an early resistant response [26], which is supported by the finding that the number of genes differentially expressed at 96 HPI, in the susceptible cultivar is more comparable to the number seen at 24 HPI in the resistant cultivar (Figure 2A). It is important to note that given the small number of differentially expressed genes and relatively smaller magnitude of differential expression seen in the susceptible cultivar compared to the resistant, no genes met the false discovery rate (FDR) correction cut-off after infection in the susceptible cultivar (see Materials and Methods for details). Thus, these differentially expressed genes must be viewed with caution. Nevertheless, it was seen that 325 were up-regulated and 60 genes were down-regulated, in response to *Xoo* infection in both the resistant and susceptible cultivars (Figure 2A), excluding genes up-regulated at one time and down-regulated at another time. Thus, in terms of timing, number and magnitude, the transcriptomic response in the resistant cultivar was greater than the susceptible cultivar in response to *Xoo* infection.

In order to identify any relation between the observed expression and function of the encoded genes, Pageman over-representation analysis was carried out [27]. Overall, it was evidenced that from 24 HPI, genes encoding photosynthesis components were down-regulated and genes encoding stress response functions were up-regulated in both the resistant and susceptible cultivars (yellow boxes; Figure 2B). The down-regulation of genes encoding major CHO metabolism, cell wall, secondary metabolism functions and specific TF families, was seen to be a unique response only to resistant infection (green boxes; Figure 2B). Similarly, the up-regulation of lipid metabolism, secondary metabolism functions and down-regulation of redox and polyamine metabolism was largely seen only in the response to infection in the susceptible cultivar. Interestingly, in the resistant cultivar, genes encoding signalling, RNA processing, RNA binding, NAC and WRKY TFs were seen to be significantly over-represented, as well as translations functions including genes encoding ribosomal proteins (green boxes; Figure 2B). A recent study analysed the transcriptomic response to bacterial infection with *Xanthomonas oryzae* pv. *oryzicola* (*Xoc*) in a susceptible cultivar, and when these microarrays were analysed in parallel to those shown here (Materials and Methods; Table 1), it was seen that the transcriptomic response showed some conservation to that seen in response to *Xoo* infection in this study, including the up-regulation of pathogen related stress responsive genes and down-regulation of photosystem I components (overlapping over-representation is indicated in purple boxes; Figure 2B). In addition, the down-regulation of genes encoding cellulose synthesis functions, isoprenoid metabolism, yabby transcription factors (TFs) and sulphate transporters was also seen under both *Xoo* infection (this study) and *Xo*c infection [16] (Figure 2B), suggesting common transcriptomic responses under these bacterial infections. Overall, the analyses carried out in this study have confirmed features seen in previous studies, and identified additional responses in the resistant compared to susceptible cultivars.

**Table 1:** Overview of the Affymetrix rice genome microarrays used for the analysis in this study

**Sample details**	**GEO accession**	**Rep**	**No**. **arrays**	**Tissue**	**Publications**
**Biotic stress - Rice**					
*X.oryzae* pv o*ryzae* bacterial infection cv. IRBB21 (resistant), IR24 (susceptible)	GSE43050	3	24	Leaf	This study
Infection with *X.oryzae* pv. oryzicola bacterial infection cv. Nipponbare	GSE19239	3	6	Leaf	[16]
*S.Hermonthica* plant parasite infection cv. Nipponbare (resistant), IAC165 (susceptible)	GSE10373	2	24	Root	[14]
*M.grisea* blast fungus infection cv. Nipponbare	GSE7256	2	8	Leaf	[13]
*M.oryzae* fungus infection cv. Nipponbare	GSE18361	2	6	Root	[19]
Rice stripe virus infection cv. WuYun3, KT95-418	GSE11025	3	12	Seedling	-
**Abiotic stress** - **Rice**					
Drought, salt, cold stress cv. IR64	GSE6901	3	12	Seedling	[17]
Heat stress cv. Zhonghua 11	GSE14275	3	6	Seedling	[18]

The microarray experiments are classified as biotic and abiotic stress. For each microarray dataset, a brief experimental description is given with the respective cultivar (cv.) indicated, reference (where available), public Gene Expression Omnibus (GSE) identifier or MIAME Genexpress identifier (E-MEXP), the number of biological replications carried out (Reps), the number of arrays carried out in that experiment and the tissue analysed are shown.

### Common and distinct transcriptomic responses to biotic stress

**Figure 3:**
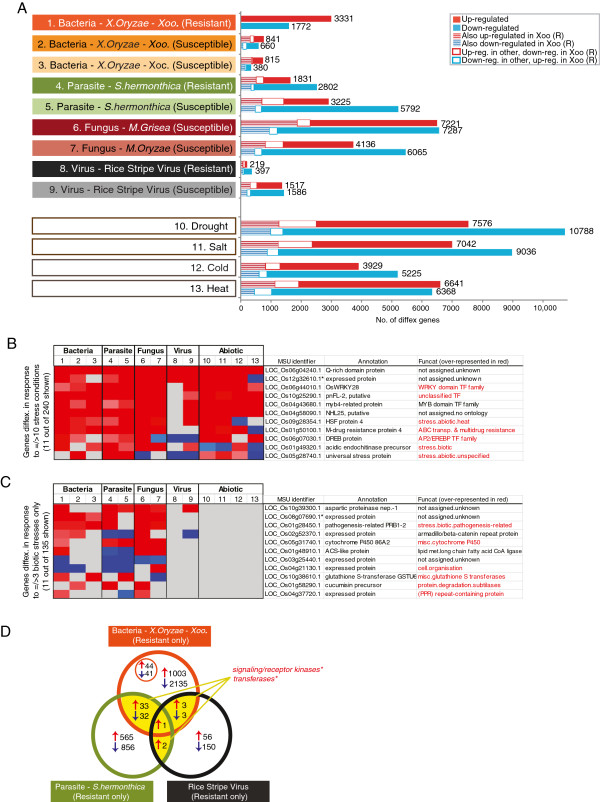
**Identification of markers of biotic stress and resistance. A**) The number of differentially expressed genes in response to biotic and abiotic stresses. The number of these overlapping with resistant response to Xoo. infection are indicated in the striped sub-sections. **B**) Identification of novel “universal stress responsive” genes. Genes showing differential expression across the most number of stress conditions are shown, as well as example genes for over-represented functional categories in this set (z-score analysis; p<0.05). **C**) Genes that were differentially expressed only in response to biotic stress, as well as example genes for over-represented functional categories in this set (z-score analysis; p<0.05). **D**) Venn diagram showing the overlap in resistant responses to bacterial infection (this study), parasite [14], and viral infection (GSE11025). Note that the genes in these subsets were only counted if the gene was not differentially expressed in the susceptible cultivar i.e. resistant response only.

In order to investigate the overlap in transcriptomic responses to stress, nearly 100 microarrays analysing responses to infection with Bacteria, Xoo – this study; Xoc - [16]; parasite, *S.hermonthica* - [14]; virus – GSE11025; fungus, *M.grisae* - [13], *M.oyzae* – [19] and abiotic stress – [17] were examined (Table 1, Additional file 1: Table S2). In this way, it was possible to determine the number of genes differentially expressed in response to each stress, the number of those that overlap with the response to resistant infection to *Xoo* (striped blocks; Figure 3A), and the number that differed to *Xoo* (open boxes; Figure 3A). It can be seen that the largest number of genes that showed a common response to *Xoo* infection was for the genes responding to susceptible leaf infection with *M.grisea*[13], where 3093 genes showed overlapping responses (Figure 3A). Similarly, the response to drought (2276 genes overlapping) and salt (2175 genes overlapping) also showed large overlapping responses with resistant infection to *Xoo*. However, it is important to note that the overall number of differentially expressed transcripts in response to *M.grisea*, drought and salt were more than double the number of transcripts responding to bacterial infection with Xoo (Figure 3A) indicating that these only represent a small proportion of the total transcriptomic response to these stresses.

Given that all of these microarrays were analysed in parallel (Table 1), it was also possible to identify highly stress responsive genes across both biotic and abiotic stresses and in this way, a shortlist of 240 genes were identified that were differentially expressed across 10 or more stresses (Additional file 1: Table S3; Figure 3B). These genes were analysed for over-represented functional categories and it was seen that these were enriched in genes encoding WRKY TFs, AP2/EREBP TFs and unclassified TFs, example genes from these categories are shown in Figure 3B (over-represented functional categories are shown in red font). In addition, as expected, ABC transporters and multidrug resistance functions encoding proteins as well as other “stress response” categories were also seen to be highly significantly enriched in this geneset, examples of these genes such as LOC_Os5g28740.1 (annotated as a universal stress protein) are shown in Figure 3B). Interestingly, of the 240 genes, 42 genes are annotated as “expressed protein” (i.e. no annotated function) with 6 of these encoding rice lineage specific genes (Additional file 1: Table S3; [28]). Given that the genes and functional categories seen in this geneset represent a significant over-representation of functions that are highly characteristic of the plant stress response, it is very likely that these 42 genes (of unknown function) represent crucial proteins involved in the abiotic and biotic stress response. This is particularly likely given that a number of proteins, such as those encoding WRKY and NAC TFs within this dataset represent genes that are known to result in increased sensitivity to stress when these genes are knocked-out/overexpressed [10,20-22].

Although more genes showed overlapping responses (striped boxes; Figure 3A) than opposite responses (open boxes; Figure 3A) across the different stresses, it can be observed that some genes were up-regulated under bacterial infection and down-regulated under other stresses e.g. an AP2 transcription factor (LOC_Os06g07030.1; Figure 3B) which is highly up-regulated under bacterial, parasite and fungal infection and is down-regulated in response to viral infection (Figure 3B). Similarly, two genes encoding an acidic endochitinase (LOC_Os1g49320.1; Figure 3B) and chitinase 1 (LOC_Os10g28080.1) were up-regulated under biotic infections, and down-regulated in response to abiotic stress (Figure 3B). In addition, specific kinases (e.g. the protein kinase - LOC_Os04g44910.1), was seen to be induced in response to bacterial, parasite, fungal and viral infection, whilst significant down-regulation was observed in response to cold and heat stress. Given that these experiments were carried out in different laboratories, it is acknowledged that antagnostic responses may be due to experimental design, and thus in defining these criteria, only genes that were significantly up-regulated in one stress and down-regulated in another or *vice versa* were defined as antagonistic. Notably, one gene was up-regulated across all 13 stresses and this is annotated to encode a Q-rich domain containing protein (LOC_Os06g04240.1; Figure 3B). Interestingly, the second most differentially expressed gene (LOC_Os12g32610.1; Figure 3B), encodes a gene with no known functional information to date, however this gene has been previously defined as lineage specific [28], i.e. only present in rice and therefore could not be identified by orthology (Figure 3B).

Given that these analyses have examined the transcriptomic response to a number of biotic and abiotic stresses in parallel, it was possible to use this data to identify genes that were only responsive to biotic stress. In this way, 135 genes that were differentially expressed across 3 or more biotic stresses (and were unresponsive to abiotic stress) were identified (Additional file 1: Table S4). These were also examined for over-represented functional categories and it was seen that there was an enrichment of genes encoding protein degradation functions, specifically subtilases, cytochrome p450 components, glutathione-S-transferases, PPR repeat containing proteins, proteins involved in cell organisation and as expected, proteins annotated as encoding pathogenesis related functions (Figure 3C). The expression of the top (biotic stress only) responsive genes is shown, as well as examples of genes from over-represented functional categories in Figure 3C. Interestingly, of the 135 genes, 25 genes are annotated as “expressed protein” (i.e. no annotated function) with 2 of these encoding rice lineage specific genes (Additional file 1: Table S4; [28]). For example the lineage specific rice gene LOC_Os08g07690.1, was seen to be up-regulated specifically in response to bacterial, parasite and fungal infections independently, whilst not responding to abiotic stress, suggesting a fundamental role in biotic stress response that is specific to rice (Figure 3C).

It is evidenced that there are overlaps in the transcriptomic responses to various biotic stresses (Figure 3), however, it is unknown how well conserved the transcriptomic response to resistance is across these different biotic stresses. To determine whether there is overlap in the genes responding only to resistant infection for individual biotic stresses, 74 genes (total number shaded in yellow; Figure 3D) were defined as differentially expressed only in the resistant cultivars (and not differentially expressed in the susceptible cultivars) to the three biotic stresses (bacteria, *Xoo*; parasite, *S.hermonthica*; virus, rice stripe virus; Figure 3D). Of these, a significant enrichment (p<0.05) of signalling/receptor kinases and transferases were seen for the up-regulated genes, whilst the down-regulated genes were enriched in cell wall related functions and co-factor and vitamin metabolism functions, revealing that these genes may be part of a common response triggered specifically during resistant infection to bacterium/parasite(s)/virus (Figure 3D). In addition to identifying common genes responsive during resistance, a unique set of 85 genes (44 up-regulated, 41 down-regulated; Figure 3D) were also identified to be differentially expressed exclusively under resistant response to *Xoo* infection (i.e. not differentially expressed in response to stress in any susceptible cultivars, as well as in response to other biotic and abiotic stresses). Notably, it was seen that of these 85 genes (smaller orange circle; Figure 3D), 6 genes encoding protein degradation functions were down-regulated, whilst 4 genes encoding signalling functions were up-regulated. Specifically, an OsWAK (OsWAK127), a lectin-like receptor kinase, a phytosulfokine receptor precursor and a serine/threonine kinase-like protein were up-regulated exclusively under resistant *Xoo* infection (Additional file 1: Table S1). In addition to these, an NBS-LRR type putative disease resistance protein (LOC_Os02g30150.1) and a gene annotated as a resistance protein LR10 (LOC_Os04g11780.1) were also seen to be in this set (smaller orange circle; Figure 3D), exclusively responsive to resistant bacterial infection. Genes in this exclusive set represent targets that may be specifically used as markers to analyse and understand the specific aspects of *Xoo* infection.

It is important to note that although the genes identified in this study are conserved across different cultivars, the differences between cultivars can also be substantial, including substantial differences in stress tolerance. Thus, the effect of the cultivars used must be noted (Table 1) in the interpretation and application of the findings in this study.

### Cell wall and metabolism changes are characteristic of resistance

**Figure 4:**
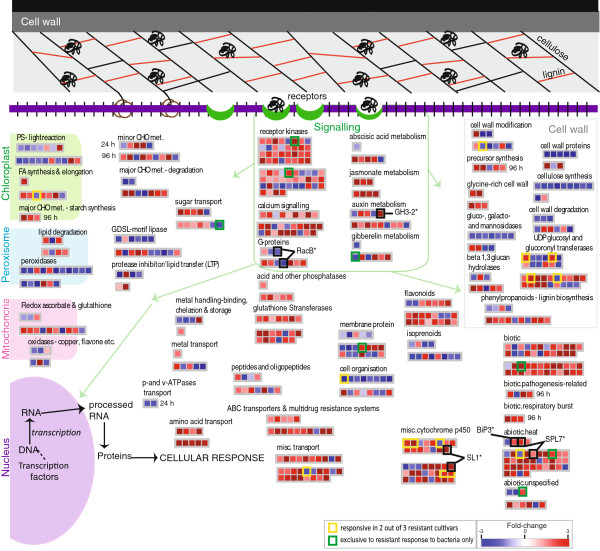
**Mapman visualisation of cell wall and metabolism functions.** The over-represented functional categories seen in the differentially expressed geneset following bacterial infection. A false coloured heatmap showing the fold-change response following Xoo. infection, where each coloured square represents the intensity of the fold change response for a single gene. Fold-changes following 24 HPI is shown above those at 96 HPI. Yellow squares indicates universal stress markers, as these genes were seen to be up-regulated following bacterial infection (this study), following parasite, fungal and viral infection [13,14], as well as following heat, cold, drought and salt stress [17,18].

**Figure 5:**
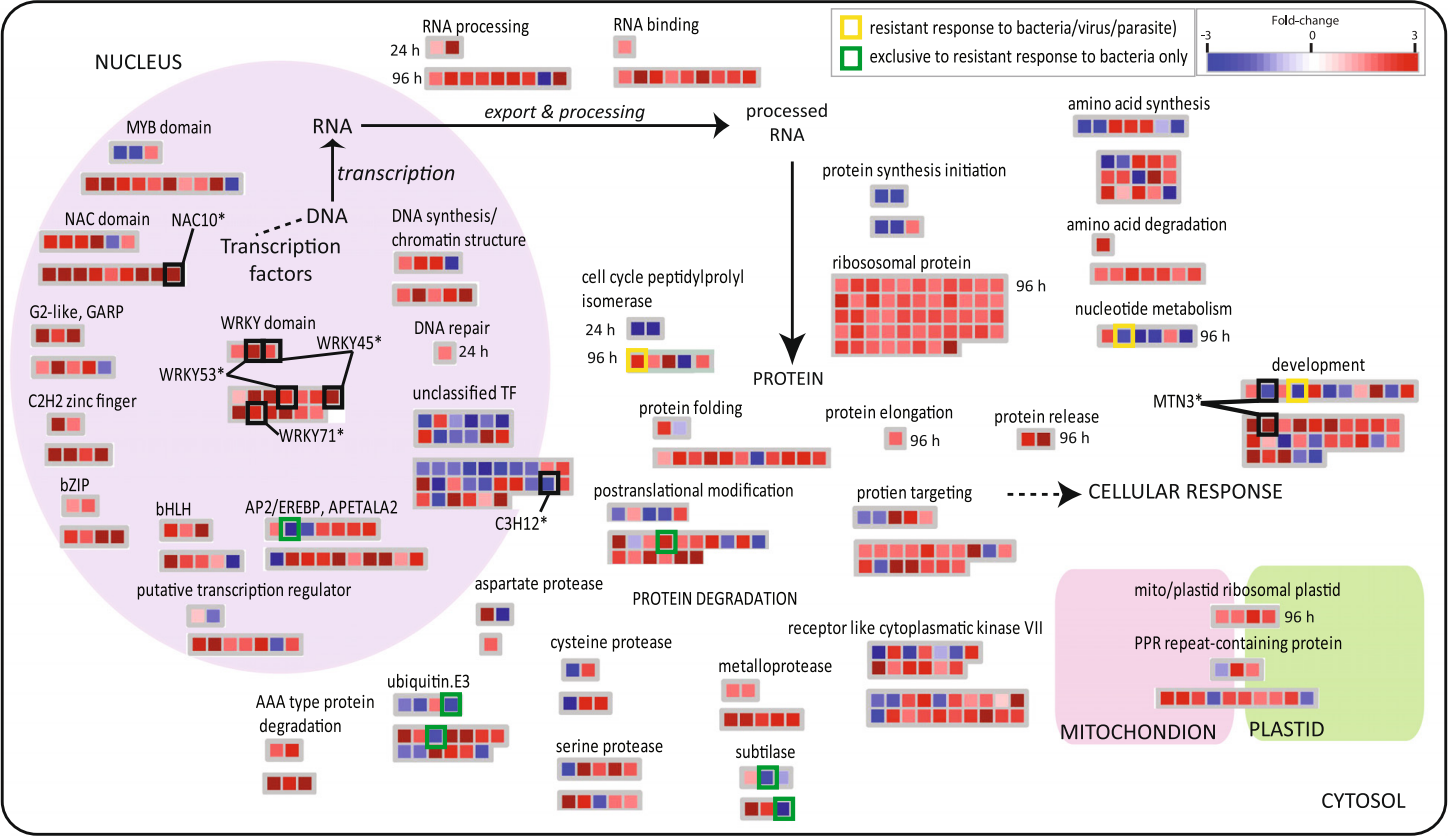
**Custom Mapman visualisation of DNA to protein targeting functions following *Xoo *infection.** A false coloured heatmap showing the fold-change response following Xoo. infection is shown, where each coloured square represents the fold change for a single gene. Fold-changes following 24 HPI is shown above those at 96 HPI. Yellow squares indicates universal stress markers, as these genes were seen to be up-regulated following bacterial infection (this study), following parasite, fungal and viral infection [13,14], as well as following heat, cold, drought and salt stress [17,18].

Overall, it can be seen that despite the 75 genes showing overlapping responses to resistant infection (Figure 3D), the response to bacterial infection in the resistant cultivar is still largely unique. Given that a significant over-representation of specific metabolic functions, signalling, transcription factors and proteins synthesis were revealed in response to resistant bacterial infection (Figure 2B), custom Mapman images were created showing the differentially expressed transcripts (p<0.05 and >1.8 fold-change) encoding cellular metabolism functions (Figure 4) and regulation and protein synthesis functions (Figure 5).

It appears that the earliest response (24 HPI) to bacterial infection in the resistant cultivar is the down-regulation of genes encoding cell wall functions, specifically those involving cellulose synthesis, with 9 of the 11 expressed genes encoding cellulose synthase showing significant down-regulation at 24 HPI in the resistant cultivar (Figure 4; Additional file 1: Table S1). In addition, genes encoding cell wall modification, cell wall degradation, cell organisation and the cell wall arabinogalactan proteins were also significantly down-regulated (Figure 4). Specifically, 5 of the 11 genes encoding fasciclin-like arabinogalactan proteins 7/8 were down-regulated at 24 HPI (Figure 4). Notably, when the differential expression of these genes was examined more closely, it was seen that while this (down-regulation) response was also common to both resistant and susceptible parasitic infection, all 5 of these genes were up-regulated in response to heat stress. Interestingly, proteins with a fascilin domain are known to play a role in cell adhesion [29], therefore the down-regulation of these in response to resistant bacterial infection (and other parasite infection) supports a role for the suppression of these functions specifically in response to biotic infections. Notably, it was seen that by 96 HPI, genes encoding proteins involved in cell wall precursor synthesis and glycine-rich cell wall structural proteins are up-regulated, suggesting that after an early down-regulation of cell wall modification and degradation functions at 24 HPI, the genes encoding cell wall structural components are then up-regulated by 96 HPI (Figure 4).

At 24 HPI, genes encoding gluco-, galacto-, mannosidases, UDP glucosyl and glucoronyl transferases, membrane proteins and secondary metabolism functions including phenylpropanoids and isopropanoids are largely down-regulated. However, by 96 HPI, an increased number of genes encoding phenylpropanoid metabolism and UDP glucosyl and glucoronyl transferases are significantly up-regulated (Figure 4). Notably, 3 of these UDP glucosyl and glucoronyl transferases encoding genes, were also up-regulated in response to resistant parasite/viral infection (genes overlapping from Figure 3D (yellow shading), boxed in yellow; Figure 4). Similarly, 2 genes encoding cytochrome 450 72A1 were also seen to be up-regulated both at 24 HPI and 96 HPI, in response to resistant bacterial infection, as well as in response to resistant parasite/viral infection (yellow boxes; Figure 4). The overlapping up-regulation of these genes in response to resistant infection suggests that these genes may have a role specifically in the resistance responses to biotic stress.

Given the significant differential expression seen for cell wall functions, it was not surprising to see differential expression of signalling functions, specifically for calcium signalling and receptor kinases. One of the differentially expressed genes encoding a receptor kinase (LOC_Os07g03920.1) was identified as exclusively up-regulated both at 24 HPI and 96 HPI exclusively in the cultivar resistant to bacterial infection (green boxes; Figure 4). Interestingly, when the expression of genes encoding kinases were compared in response to abiotic stress, it was observed that while 32 genes encoding kinases were up-regulated in response to resistant bacterial infection, 24 and 21 of these genes are in fact down-regulated in response to heat and drought and/or salt treatment, respectively, indicating that these receptor kinases are specifically responsive to biotic stress. Furthermore, 18 of the 36 genes encoding wall associated kinases (OsWAKs) were seen to be significantly up-regulated in response to bacterial infection in the resistant cultivar (receptor kinases; Figure 4). Notably, it was also seen that the expression of a Rop small GTPase gene (OsRacB), involved in signalling was down-regulated in response to infection with *Xoo* (RacB*; Figure 4). It has previously been shown that OsRacB is associated with the plasma membrane and over-expression results in increased symptoms in response to fungal (*M.grisea)* infection [30]. These expression patterns suggest a strong early response to infection in the resistant cultivar, which involves both cell wall functions and signalling.

Closer examination of genes encoding energy-related functions also revealed a down-regulation of genes encoding photosynthesis functions, peroxidases, oxidases, lipases and lipid transfer proteins, possibly indicative of an early energy conservation and defence response to infection (Figure 4). In contrast, genes encoding redox functions and fatty acid synthesis and elongation functions were up-regulated at 96 HPI (Figure 4), which is characteristic of the plant stress and defense response [31]. Specifically, it is notable that a gene encoding CYP71P1 in the cytochrome P450 monooxygenase family (annotated as SL1* in Figure 4) was highly up-regulated in response to *Xoo* infection. Interestingly, it has recently been shown that SL1 is one of the earliest genes to be significantly induced, seen as early as 1 hour after infection with the fungus *M.oryzae*[32]. It has also been shown that mutation in this gene appears to result in Sekiguchi lesion formations on rice leaves, and increased resistance to fungal infection [32,33]. Thus, the observed up-regulation of this gene suggests it may also have a role in lesion prevention/delay in the resistant response to bacterial (*Xoo*) infection.

Similarly, a large number of genes annotated as responsive to abiotic and biotic stress related functions were up-regulated in response to resistant infection to bacterial infection (Figure 4). Two of these up-regulated genes encoding heat shock factors are particularly noteworthy (denoted *SPL7 and *BiP3; black boxes in Figure 4). It has been shown that transgenic plants with suppressed SPL7 expression resulted in increased resistance to infection [34]. Similarly, the expression of an ER-located member of the heat shock protein family was up-regulated in response to bacterial infection (denoted *BiP3; Figure 4) and a recent study has shown that BiP3 overexpressing plants showed compromised Xa21 mediated immunity to *Xoo* infection [7]. Finding that suppression/over-expression of these genes results in significant changes to immunity indicates a functional role for the transcriptomic responses seen for these stress responsive genes, and suggesting that these are likely not to be the only genes that have a significant role in immunity.

### A role for RNA and protein synthesis in *Xoo* resistance

A notable over-representation of genes encoding protein synthesis, signalling and specific transcription factors including those encoding NAC and WRKY TFs were observed to be up-regulated in the resistant response to bacterial infection (Figure 2B; Figure 5). Interestingly, an examination of the individual gene expression levels revealed a significant up-regulation of RNA functions; specifically TFs, RNA processing, RNA binding, ribosomal proteins, protein folding and targeting at 96 HPI (Figure 5). Closer examination of these genes showed that of the 50 genes encoding ribosomal proteins that were up-regulated in the resistant response to bacterial infection (Figure 5), 46 of these were also up-regulated in response to fungal infection in leaves (*M.grisea*; Table 1). The significant up-regulation of these in response to both bacterial and fungal infection, suggests these functions may be are actively required in response to these infections. However, 42 and 46 of these 50 ribosomal protein encoding genes were significantly down-regulated in the response to parasite infection (susceptible cv. IAC) and in response to both drought and salt treatment, respectively. Furthermore, it was seen that while 10 genes encoding heat shock proteins were up-regulated in response to resistant bacterial infection, 9 of these were also up-regulated in response to fungal infection (*M.grisea*), all 10 genes were down-regulated in response to root fungal infection with *M.oryzae*. These distinct responses indicate that while the up-regulation of specific functions e.g. translation appears to be a common response to resistant bacterial infection and in response to fungal infection with *M.grisea*, the opposite is observed in the susceptible response to parasite infection and under drought and salt stress.

A particularly interesting finding in the resistant response to bacterial infection was the over-representation of genes encoding cell cycle peptidyl isomerases. It can be seen at although 2 genes encoding cell cycle peptidyl isomerases were down-regulated at 24 HPI, 4 genes are up-regulated by 96 HPI with one of these genes (LOC_Os01g38359.1) even seen to be only up-regulated in the resistant responses, to both bacterial (at 96 HPI) and parasite infection (yellow square; Figure 5). Although only 7 differentially expressed genes encoding cell cycle peptidyl isomerases are shown in Figure 5, another 8 genes encoding cell cycle peptidyl isomerases were also significantly up-regulated at 96 HPI, however these were up-regulated by 1.4-1.8 fold and therefore are not displayed. The finding that a significant over-representation i.e. 15 out of the 41 genes encoding these cell cycle isomerases were differentially expressed (known to be involved in protein folding [35] suggests a specific role for these genes in the resistant response to bacteria.

Interestingly, it was seen that while RNA processing and translation function were up-regulated in response to resistant infection with bacteria, nucleotide and protein degradation functions including genes encoding proteins in the ubiquitin E3 complex and subtilises were down-regulated in the resistant response to bacterial infection (Figure 5). Notably, genes encoding a speckle-type POZ protein (LOC_Os10g29220.1) as part of the ubiquitin E3 complex and a subtilisin-like protease precursor (LOC_Os04g02970.1) were seen to be down-regulated exclusively in the response to bacterial infection in the resistant cultivar (green boxes; Figure 5), whilst a gene encoding a protein deaminase involved in nucleotide degradation (LOC_Os07g46630.1) was down-regulated in response to both bacterial and parasite infection in the (respective) resistant cultivars only (yellow box; Figure 5). Interestingly, several genes encoding functions annotated as involved in development were differentially expressed (Figure 5). Notably, one of these genes encoding a member of the MTN3/saliva gene family (also known as xa25) has previously been shown to have a role in race-specific resistance to *Xoo* infection [36]. Interestingly, this MTN3 gene was first down-regulated at 24 HPI, before being significantly induced at 96 HPI (*MTN3; black box in Figure 5). It has been suggested that this protein may have a role in sugar transport in response to *Xoo* infection in rice [36], and the observed opposite changes in expression seen at 24 and 96 HPI (Figure 5) suggests that the expression of this gene may be tightly controlled in response to *Xoo* infection.

In response to infection, a number of unclassified TFs were seen to be down-regulated (Figure 2B; Figure 5). Upon closer examination of these genes, it was seen that a number of these TFs have been shown to have a direct functional role in response to infection/stress. One of these genes includes a zinc finger family protein - C3H12, which has recently been shown to be involved in rice resistance to *Xoo* infection, with knock-out of this gene seen to result in partially increased susceptibility to *Xoo* infection in Zhonghu 11 [37]. Upon bacterial infection in this study, C3H12 did not change in expression at 24 HPI, however, it was seen to decrease in expression at 96 HPI (−1.8 fold; C3H12*, black box in Figure 5). In contrast, some of the most strongly up-regulated genes encoding regulatory functions included those encoding NAC and WRKY TFs (Figure 5). Specifically, 3 WRKY TFs that were significantly up-regulated (>1.8 fold), have been shown to result in altered resistance when knocked-out or overexpressed (black boxes; Figure 5). It has been shown that over-expression of WRKY53 and WRKY71 resulted in enhanced resistance to fungal infection (*M.grisea;*[38] and bacterial infection (*Xoo*; [23]), respectively. In contrast, plants over-expressing WRKY45 have been shown to result in increase susceptibility to bacterial infection (Xoo; [10]). This suggests a functional role for the up-regulation of these genes encoding TFs in response to infection. Interestingly, previous studies analysing the role of NAC TFs have mainly focussed on a role for these TFs in development and the response to abiotic stresses [10,20,22,39,40]. For example, NAC10 has been shown to result in a 53-fold induction in response to drought stress [17] and over-expression has been seen to result in root enlargement and increased resistance to drought stress [20]. However, it is likely that NAC10 and a number of the other NAC TFs seen to be induced in this study also have a role in biotic stress resistance, with one of these genes encoding NAC18 (LOC_Os07g48450.1) showing a 40-fold up-regulation at 96 HPI in response to bacterial infection (Figure 5).

Furthermore, given that a number of these NAC TFs are seen to be up-regulated, even as early as 24 HPI, all up-regulated genes in response to bacterial infection in the resistant cultivar were examined for over-represented putative *cis*-acting elements in the 1kb upstream regions of these genes. To do this, the 1kb upstream DNA sequences of this sub-set were extracted and the occurrence of all motifs shown in AGRIS and Athamap [41,42] were calculated for each subset, shown as a percentage, and compared to the percentage occurrence of each motif across all 1kb upstream sequences in the rice genome (see Methods). All significantly (p<0.05) over-represented known elements (as defined in AGRIS and Athamap; [41,42]) are shown in Table 2. Interestingly, it was seen that for the genes responding to bacterial infection in the resistant cultivar, the number of up-regulated genes that contained Abscisic acid Response Elements (ABREs), Heat Shock Elements (HSEs) and NAM, ATAF, and CUC (NAC) elements was significantly higher than expected (Table 2; z-score, p<0.05). This was particularly obvious for genes containing putative NAC binding sites, given that 10 different NAC elements, with a CACG core, were seen to be over-represented in the promoters of these up-regulated genes, with one of these elements, CACG(T/C)A, even enriched in the upstream regions of both the up-regulated and down-regulated sets of genes (Table 2). Notably, it was seen that when the up-regulated genes containing HSEs were extracted and examined, it was evidenced that 15 of the 30 genes annotated as responsive to heat stress (abiotic.heat.stress) contained the known HSE; GAAGCT [41]. Similarly, when the genes that contained TELOBOX elements were examined, it was evidenced that 32 out of the 50 ribosomal proteins contained these TELOBOX elements (Table 2). As seen for the HSEs, it is known that the promoter regions of ribosomal proteins are enriched in TELOBOX elements [43], thus it is possible that at one or more elements presented in Table 2 represent functional TF binding sites.

**Table 2:** **Over**-**represented putative motifs**/**binding sites in the 1kb upstream promoter region of genes down**/**up**-**regulated in response to *Xoo***

**Final motif**^**Source**^	**Name**	**Genome (35,811)**	**Down-reg. (514)**	**Up**-**reg. (1163)**
ACGTGG^**1**^	ABRE/GBF1/2/3	6725 (**19**%)	76 (**15**%)	241 (**21**%)
CACGTG^**1**/**2**^	ABRE/G-box/NAC	6701 (**19**%)	91 (18%)	251 (**22**%)
CGTGTA^**1**^	ABRE-like	4266 (**12**%)	63 (12%)	164 (**14**%)
ACACGT^**1**^	ACE	5764 (**16**%)	87 (17%)	239 (**21**%)
GACACG^**1**/**2**^	ACE/NAC	5249 (**15**%)	69 (13%)	221 (**19**%)
CCACGT^**1**^	CBF2/GBF1/2/3	7265 (**20**%)	103 (20%)	289 (**25**%)
AAAAAT^**1**^	CCA1 2 BS in CAB1	24552 (**69**%)	357 (69%)	836 (**72**%)
(T/C)TCCCG^**1**/**2**^	E2F	6842 (**19**%)	89 (17%)	250 (**21**%)
(T)CCCGC(C )^**1**^	E2F	5779 (**16**%)	79 (15%)	215 (**18**%)
AGCCGC^**1**^	ERE/ERF	6679 (**19**%)	81 (**16**%)	248 (**21**%)
(A)GAAGC(T)^**1**^	HSEs	7037 (**20**%)	108 (21%)	261 (**22**%)
(A)ACGTT(C )^**1**^	HSEs	2792 (**8**%)	47 (9%)	116 (**10**%)
CGTTCT^**1**^	HSEs	4198 (**12**%)	70 (14%)	163 (**14**%)
(A)GCTTC(T)^**1**^	HSEs	7588 (**21**%)	121 (24%)	297 (**26**%)
GATCGA^**1**^	Nonamer	9377 (**26**%)	137 (27%)	344 (**30**%)
AAACCC^**1**^	TELO-box	10238 (**29**%)	148 (29%)	404 (**35**%)
TACGTG^**1**^	ABRE/Z-box	5252 (**15**%)	73 (14%)	198 (**17**%)
AGCCGT^**2**^	AtERF-3	4565 (**13**%)	70 (14%)	172 (**15**%)
(A)CGTGT(C )^**2**^	ABI5/bZIP	5508 (**15**%)	86 (17%)	224 (**19**%)
ACGTAG^**2**^	ABI5/bZIP	3974 (**11**%)	50 (10%)	149 (**13**%)
TTAGTT^**2**^	AtMYB44	11540 (**32**%)	161 (31%)	412 (**35**%)
CACGT(T/C )^**2**^	NAC	4410 (**12**%)	63 (12%)	176 (**15**%)
(G/T/A)CCACG^**2**^	NAC	5583 (**16**%)	75 (15%)	220 (**19**%)
(C/A)ACACG^**2**^	NAC	5418 (**15**%)	84 (16%)	226 (**19**%)
GTCACG^**2**^	NAC	4111 (**11**%)	62 (12%)	157 (**13**%)
CACGCG^**2**^	NAC	6573 (**18**%)	80 (16%)	267 (**23**%)
CGCACG^**2**^	NAC	6325 (**18**%)	89 (17%)	230 (**20**%)
CACGAA^**2**^	NAC	5705 (**16**%)	86 (17%)	218 (**19**%)
(A)TGCAT(T)^**2**^	ABI3	9558 (**27**%)	162 (**32**%)	297 (26%)
ATGCAA^**2**^	ABI3	10545 (**29**%)	172 (**33**%)	335 (29%)
CACGAG^**2**^	NAC	5604 (**16**%)	99 (**19**%)	186 (16%)
AATGCA^**1**^	L1-box	8984 (**25**%)	149 (**29**%)	289 (25%)
GAAAAA^**2**^	GT-3b	21164 (**59**%)	328 (**64**%)	706 (61%)
CACATG^**1**^	AtMYC2 BS in RD22	10380 (**29**%)	172 (**33**%)	312 (27%)
(G)CAACA(G)^**1**^	RAV1-A	8308 (**23**%)	151 (**29**%)	273 (23%)
CGTACA^**1**/**2**^	SBP-box/SPL	3940 (**11**%)	77 (**15**%)	138 (12%)
(T)CAAGT(G)^**1**^	SORLIP3	7327 (**20**%)	123 (**24**%)	247 (21%)
TGACGA^**2**^	WRKY18	6025 (**17**%)	107 (**21**%)	193 (17%)
AAGCTT^**1**^	HSEs	7981 (**22**%)	141 (**27**%)	295 (**25**%)
CACG(T/C)A^**2**^	ABI5/NAC/bZIP	6626 (**19**%)	118 (**23**%)	260 (**22**%)

Source: 1= [41,42], 2=[41,42].

Infection in rice.

### Functional roles for the transcriptomic responses

In order to establish a functional link between the responses observed in this study and resistance to *Xoo* infection, an extensive literature search was carried out mining any publications that presented data on Xoo/X*anthomonas oryzae pv. oryzae* infection in rice. In this way, 54 genes were found, which were both expressed in the leaf tissues in this study, and were identified to have a functional role in stress resistance, given that resistance was directly altered upon knock-down/knock-out or over-expression of these genes. Interestingly, 33 of these genes were significantly differentially expressed in response to Xoo infection in this study, suggesting that these likely represent genes that are not only regulated at a transcript level in response to stress, but also have a functional role in altering resistance (Table 3). The finding that suppressing/over-expressing these genes have been shown to result in altered resistance phenotypes (Table 3) suggests that the response to infection, whether resistant or susceptible involves significant regulation at the transcript level.

**Table 3:** **Differentially expressed genes encoding proteins shown to have a role in biotic**/**abiotic stress resistance**, **when knocked**-**out** (**KO**)/**over**-**expressed** (**Ox**)

**Probe Set ID**	**Bacteria**			**Parasite**		**Fungus**		**Virus**		**Abiotic**				**MSU gene identifier**	**Name**	**Phenotype**	**Reference**
	**1**	**2**	**3**	**4**	**5**	**6**	**7**	**8**	**9**	**10**	**11**	**12**	**13**				
Os.11773.1.s1_at	2.1			2.8	2.3	9.2	7.1	2.0		2.4	2.4	2.3	−2.0	LOC_Os05g27730.1	OsWRKY53	Ox - enhanced resistance	[38]
Os.12032.1.s1_at	2.0		2.9	8.3	8.5	80.7	25.5	2.2		3.4	2.8	4.8		LOC_Os02g08440.1	OsWRKY71	Ox - enhanced resistance	[23]
Os.37565.2.s1_at	2.8			11.3	2.9	6.5	4.7	2.2		1.3		8.3	−1.9	LOC_Os05g25770.1	*OsWRKY45-1*	Ox - increased susceptibility	[10]
Os.48082.1.s1_at	1.6	6.1		15.3		25.8		12.0		2.2	9.2	2.2		LOC_Os09g25070.1	OsWRKY62	Ox - increased susceptibility	[44]
Os.50015.1.s1_at	1.5	2.1	7.0	46.7	21.0	219.4	22.3	4.6		8.9	44.5	7.7	1.7	LOC_Os06g44010.1	OsWRKY28	Ox - enhanced resistance	[21]
Os.2160.2.s1_x_at	1.4		1.6		2.1	1.6	−1.5			1.8	1.8		−2.5	LOC_Os01g54600.1	OsWRKY13	Ox - enhanced resistance	[11]
Os.15708.1.s1_a_at	1.2						−3.4				2.2	1.7	−9.2	LOC_Os04g38720.1	OsNAC2	Ox- increased shoot branching	[39]
Os.4385.1.s1_at	1.5		1.9		1.4	1.9	−1.7			11.1	8.7	2.2	1.7	LOC_Os11g08210.1	OsNAC5	Ox-increase stress tolerance	[22]
Os.12199.1.s1_at	1.4			1.7	1.6	8.9				6.9	5.7	3.4	1.4	LOC_Os01g66120.1	OsNAC6	Ox-increase stress tolerance	[22]
Os.26695.1.s1_at	1.6		2.0	2.7	3.4	55.6	4.0	3.0		7.0	5.3	4.9	5.9	LOC_Os03g60080.1	NAC9/SNAC1	Ox-increase stress tolerance	[40]
Os.35020.1.s1_at	4.1	11.1	2.2	3.9	4.1	112.1				53.1	35.1	3.3		LOC_Os11g03300.1	NAC10	Ox-increase stress tolerance	[20]
Os.25621.2.s1_at	10.0	3.9	3.4	10.7	8.1	65.1	15.5			9.5	6.0		4.6	LOC_Os12g16720.1	SL1	KO/KD - enhanced resistance	[33]
Os.12372.2.s1_x_at	−1.4		−2.4	−1.5	−2.1	−3.4	−1.8			−5.9	−3.4	−3.1		LOC_Os08g06280.3	OsLSD1	Ox - increased susceptibility	[45]
Os.42024.1.s1_at		−1.7			1.6	−4.0	2.2			−2.0	−2.3	1.8	−15.2	LOC_Os01g56420.1	COPT1	Ox - increased susceptibility	[46]
Os.40018.1.s1_at	2.0			6.1	2.1	36.5	54.5	2.6			4.0	1.9		LOC_Os05g45410.1	SPL7	KO/KD -increased susceptibility	[34]
Os.12767.1.s1_a_at	−1.5			−1.7		22.0	−2.3	−2.5		1.5	1.5		−2.3	LOC_Os07g34570.1	OsDR8	KO/KD -increased susceptibility	[47]
Os.12501.1.s1_at	8.0				1.6	66.7	−2.1				6.0	−2.0	−4.5	LOC_Os01g55940.1	GH3-2	Activation - enhanced resistance	[48]
Os.11798.1.s1_at	1.7		10.8				8.5			11.7	2.8	1.7	1.7	LOC_Os07g40290.1	OsGH3-8	Ox - enhanced resistance	[49]
Os.2448.1.s1_at	−2.7			−2.1	−2.2	2.5	−1.4			−1.6	−1.8		4.4	LOC_Os02g02840.1	OsRacB	Ox - increased susceptibility	[30]
Os.4684.1.s1_at	1.6				−1.8	4.5				−2.0	−2.3		1.4	LOC_Os01g49290.1	Rack1	Ox - enhanced resistance	[50]
Os.10401.1.s1_s_at		14.5				46.2						−1.8		LOC_Os08g42350.1	xa13/8N3	KO/KD - enhanced resistance	[51]
Os.5491.1.a1_s_at	2.4	2.4	−2.3			4.5	−1.5			1.2	1.6			LOC_Os12g29220.1	MtN3/saliva	Expression - enhanced resistance	[36]
Os.27244.1.a1_s_at	−7.3		−4.9			−3.3				−1.4	1.2			LOC_Os04g32850.1	Pi21	KO/KD - enhanced resistance	[52]
Os.1311.1.s1_at	2.9					4.0				−2.3	−1.6	−1.5		LOC_Os02g02410.1	BiP3	Ox - increased susceptibility	[53]
Os.19321.1.s1_at	1.4				1.9	6.4	5.1	3.5			1.4	1.7	−1.8	LOC_Os03g60650.1	XB15	KO/KD - enhanced resistance	[54]
Os.8901.1.s1_at	1.6			1.8	2.3	3.1	2.9					2.6		LOC_Os02g22130.1	OsGAP1	Ox - enhanced resistance	[55]
Os.9338.1.s1_at	1.4					1.9				1.2	1.2			LOC_Os01g43540.1	OsSGT1	Ox - enhanced resistance	[56]
Os.8353.1.s1_at		2.8					−1.9	−3.0		4.3	3.9	1.9	2.2	LOC_Os01g68770.1	OsSBP	Ox - enhanced resistance	[57]
Os.27112.1.s1_at	1.7			1.9	1.8	12.7				2.5	3.2		−5.1	LOC_Os01g09800.1	NH1	Ox - enhanced resistance	[58]
Os.19086.2.s1_x_at	−1.8				−1.4	1.5				1.3			1.7	LOC_Os01g68860.1	C3H12	KO/KD -increase susceptibility	[37]
Osaffx.19417.2.s1_x_at		−1.1												LOC_Os11g47210.1	xa26	Ox - enhanced resistance	[59]
Os.406.1.s1_a_at	1.7		2.2	1.8		19.7	9.0	2.7	2.6	2.9		3.2	−3.4	LOC_Os03g17700.1	BIMK1/OsMPK5	KO/KD - enhanced resistance	[60]
Os.6126.1.s1_at		−1.6				3.8	3.6	3.2		1.4	3.2	1.9	2.1	LOC_Os04g41160.1	OsOxi1 (90%)	Ox - enhanced resistance	[61]

The Affymetrix probeset ID, fold-change responses to: Bacterial infection; 1. Resistant (Xoo.), 2. Susceptible (Xoo.), 3. Susceptible (Xoc.) [16], Parasite infection [14]; 4. Resistant (*S.hermonthica*), 5. Susceptible (*S.hermonthica*), Fungus; 6. Susceptible (*M. grisea*) [13], 7. Susceptible (*M.oryzae*) [19], Virus; 8. Resistant (Rice stripe virus), 9. Susceptible (Rice stripe virus) – GSE11025, Abiotic stress [17]; 10. Drought, 11. Salt, 12. Cold, 13. Heat [18]. In addition, the locus identifier (MSU), gene name, phenotype and reference are also shown for each gene.

Upon examination of these, it is immediately apparent that nearly all of these genes were significantly responsive at the transcript level, not only in response to bacterial infection (as seen in this study), but also in response to a number of other biotic stresses including; parasite infection e.g. *Striga hermonthica*[14], fungal infection e.g. *Magnoporithea grisea, Magnoporithea oryzae*[13,19], viral infection e.g. Rice Stripe Virus (RSV) and abiotic stresses, such as drought, salt, cold and heat [17,18] (Table 3). Each of these individual studies [13,14,17-19] each gave insight into the plant defence response to a specific stress. However, given that plants can utilise similar defence strategies in response to different stresses, and are usually exposed to more than one stress in at a time, or over the course of the plant lifetime, it is not unexpected that the plant defence response has evolved to generate common and distinct responses that allow both flexibility and specificity of the stress response. For example, 4 genes encoding WRKY TFs were significantly up-regulated in response to bacterial infection (and in response to other stresses; Table 3) and have been shown independently to result in increased resistance to bacterial and/or fungal infection when these genes were over-expressed [11,21,23,38], with the over-expression of WRKY13 seen to result in increased resistance to both bacterial (*Xoo*) and fungal (*M.grisea*) infection (Table 3) [11]. Similarly, 4 genes encoding NAC TFs were induced in response to several biotic and abiotic stresses, with over-expression of these also seen to result in increased salt and/or drought tolerance (Table 3) [20,22,39,40]. Apart from genes encoding TFs, a number of genes encoding signalling functions including G-proteins (GH3-2, OsGH308, OsRacB and Rack1) and kinases (Xa26, OsMPK5) have also been shown to have a functional role in response to infection (Table 3). Notably, 30 out of the 33 genes were significantly differentially expressed in response to 5 or more stresses, with 6 of these genes differentially expressed in response to 10 or more stresses (i.e. within the set of 240 genes identified in Figure 3B). Thus, the genes identified in this study as responsive to multiple stresses (Figure 3; Additional file 1: Table S1) represent genes highly likely to be functional in stress resistance.

## Discussion

**Figure 6:**
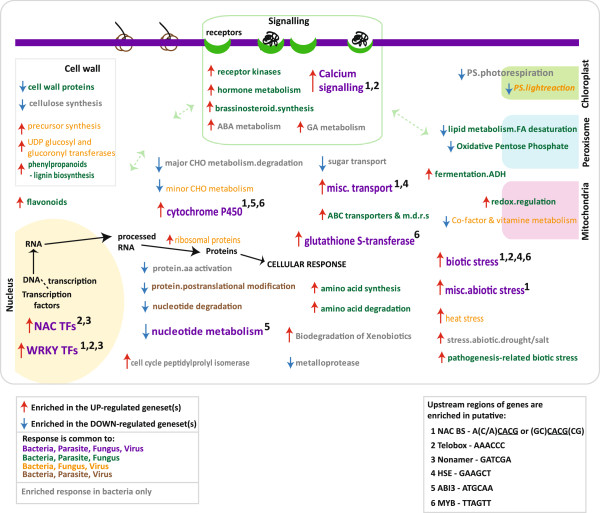
**Transcriptomic signatures of biotic stress responses.** Pageman analysis was carried out revealing the over-represented functional categories across the differentially expressed genes following for bacterial, parasite, fungal and viral infections. When functional categories were over-represented in the same (up/down-regulated) genesets, across 3 or more biotic stresses, these functional categories are shown with the common response (up/down) indicated by a red/blue arrow, respectively. In addition to these, the functional categories seen only to be responsive under bacterial infection are indicated in grey. Also, over-represented putative motifs (listed in Table 2) for the genes in each of the overlapping, over-represented functional categories are shown, with specific motif(s) indicated by a number(s).

By examining the expression patterns of genes in response to a range of biotic stresses (and abiotic stresses) in parallel, for the first time, it was possible to develop an outline or model showing similarities in the transcriptomic responses to biotic stress including infection with bacterium (this study), fungus [13,19], parasite [14] and virus (GSE11025). Pageman over-representation analysis [27] was carried out for each of these studies in parallel, and over-represented functional categories were compared for down- and up-regulated gene-sets across each stress. Figure 6 shows the over-represented functional categories, the common response to infection with bacterium, parasite, fungus and virus are shown in purple font, in green font (bacterium, parasite and fungus), orange (bacterium, fungus and virus) and brown (bacterium, parasite and virus). It appears that in response to all four biotic stresses (purple font), there is significant down-regulation of nucleotide metabolism functions and an up-regulation of genes encoding calcium signalling functions, miscellaneous transport functions, biotic and abiotic stress-response functions, glutathione S-transferases and both WRKY and NAC transcription factors (Figure 6). Studies have shown a crucial role for the induction of calcium-signalling in response to abiotic stress, with the over-expression of specific factors seen to result in increased stress tolerance in transgenic plants [62-64]. Interestingly, a recent study also showed that arbuscular mycorrhizal (root fungus) symbiosis in rice results in the induction of defence-related genes in leaves, including some crucial components involved in calcium signalling, ultimately resulting in resistance to pathogen infection [65]. Therefore, it is likely that components of calcium signalling are functional in general stress recognition, including in response to biotic stress(es) (Figure 6).

In addition to calcium signalling, a conserved up-regulation of specific NAC and WRKY TFs were also evidenced across all the biotic stresses analysed (Figure 6). The importance of transcriptional regulation in response to infection is most notably evidenced by the significant alteration in resistance or stress response, seen in rice plants following knock-out/over-expression of specific NAC and WRKY transcription factors (examples shown in Table 3) [11,20-23,38-40]. These NAC and WRKY transcription factors clearly appear to not only be regulated at the transcript level in response to infection, but are also directly involved in regulating transcript abundance, given their role as transcription factors. Furthermore, apart from the genes encoding glutathione S-transferases, genes in all of the other functional categories (shown in purple font; Figure 6) contained significant enrichment of putative NAC binding elements in the promoter regions of these genes, which suggests that these genes, including the WRKY TFs, may be regulated by NAC TFs (denoted with 1; Figure 6). A recent study has even experimentally confirmed the relationship between a NAC and WRKY TF [66], supporting a conserved and related role for these TFs. Notably, a literature search was also carried out to determine the role(s) of NAC TFs in this study, and it was found that 5 NAC transcription factors that were differentially expressed in response to Xoo infection (and in response to other abiotic stresses; Table 3), have been shown result in increased stress tolerance, specifically to salt and/or drought stress, when over-expressed in rice [20,22,39,40].

Examining the transcriptomic responses to different biotic stresses in parallel this way, revealed that the response to viral infection is rather distinct from bacterial, parasite and fungal infection, with fewer functional categories showing overlapping responses (orange and brown; Figure 6). This may reflect a different mode of defence response that may more be specific to viral infection. It is evidenced that genes encoding photosystem components (light reactions) and co-factor and vitamin metabolism were also down-regulated (Figure 6). A previous study has shown that when OsDR8 (a protein involved in co-factor and vitamin metabolism) was genetically repressed in expression, increased susceptibility to bacterial (Xoo) and fungal (*M.grisea*) infection was observed [47]. Notably, this gene was seen to be down-regulated in response to bacterial infection and other biotic stresses (Table 3, Figure 6). In contrast, genes encoding ribosomal proteins, UDP-glucosyl and gluconyl transferases, heat-stress responsive proteins, and proteins involved in cell wall precursor synthesis were up-regulated, which is a conserved response following bacterial, fungal and viral infection (Figure 6). Notably, closer examination revealed that many of the heat-stress responsive proteins (Figure 6) encode heat shock factors. While heat shock factors are typically induced under heat stress [40,67] and very well known for their functional role in abiotic stress [68-70], a recent study has shown that Xa7 mediated resistance to bacterial blight (Xoo infection) was in fact more effective at high temperatures [71], indicating an possible conserved role of these factors not only in abiotic stress, but also across biotic stresses (as seen in Figure 6). In addition, a conserved down-regulation of genes encoding nucleotide degradation and post-translational modification was seen in response to bacterial, parasite and viral infection. It is important to note that although the genes or expression responses identified in this study may show conservation across different stresses in different cultivars, the basal differences between cultivars can also be substantial, including substantial differences in stress tolerance. Thus, the effect of the cultivars used must be noted (Table 1) in the interpretation and application of the findings in this study.

Overall, it is apparent that there is much overlap in the transcriptomic response to bacterial, parasite and fungal infection (green; Figure 6). This is particularly interesting for genes encoding signalling functions, with a common up-regulation seen for genes encoding receptor kinases, hormone metabolism, brassinosteriod synthesis and calcium signalling following bacterial, fungal or parasite infection (green; Figure 6). The overlap in response to bacterial and fungal infection is not unexpected, given that a number of studies have shown common defence responses to these infections. For example, activation of a gene encoding an indole-3-acetic acid (IAA)-amido synthetase (GH3-2) has been shown to result in in enhanced resistance to both bacterial (*Xoo*) and fungal (*M.grisea)* infection [72]. Similarly, OsWRKY13 overexpressing plants have also been found to result in increased resistance to both *Xoo* and *M.grisea* infection [11], while OsWRKY45 overexpressing plants were seen to result in increased susceptibility, also to both *Xoo* and *M.grisea* infection [10]. Examination of the transcript responses of these genes reveals conserved and distinct transcriptomic responses to these biotic and abiotic stresses (Table 3). For genes showing conserved, strong transcriptomic responses to multiple stresses, it is likely that these genes play a role in responses to these multiple stresses. For example, in this study, it can be seen that NAC10 is highly induced in response to 9 of the 13 stresses analysed, specifically a 53-fold induction is seen in response to drought and recent study showed that over-expression of NAC10 results in root enlargement and increased resistance to drought stress [20]. However, given that this gene is also strongly induced (>10-fold) in response to bacterial, parasite and fungal infection (112-fold in response to *M.grisea*; Table 3), it is very possible that NAC10 has a role in biotic stress resistance as well.

Interestingly, examining the bacterial transcriptomic response “in the context” of the other biotic stress responses as done in this study, has revealed that the down-regulation of genes encoding photorespiration functions, metalloproteases, sugar transport and sucrose/starch degradation (major CHO metabolism) are seen more specifically in response to bacterial infection. Similarly, the enrichment of cell cycle peptidylprolyl isomerases and ABA and GA metabolism in the up-regulated gene-sets were also only seen in response to bacterial infection (Figure 6). Given that 15 of the 41 cell cycle peptidylprolyl isomerases expressed in leaves were differentially expressed in response to bacterial infection, this suggests a role for these in the plant defense response to bacterial infection. Cell cycle peptidylprolyl isomerases have been well characterised in humans, specifically for their role in immunity [73]. These proteins are known to associate with heat shock proteins and have been shown to play a role as parasitic chaperones [74]. Their up-regulation in the rice response to bacterial infection in the resistant cultivar could suggest a role for these in the plant defence response as well.

Interestingly, 18 of the 36 OsWAKs (rice wall associated kinases) were differentially expressed during resistant bacterial infection. It has been shown that the rice genome has significantly more WAKs than *Arabidopsis thaliana* and it was revealed that this increased number is not only due to the larger genome size [75]. In fact, this study revealed that distinct groups of OsWAKs have evolved in rice, suggesting unique roles for these have evolved, that are specifically necessary only in rice (compared to Arabidopsis; [75]). Similarly, the up-regulation of genes encoding amino acid metabolism, secondary metabolism - flavonoid and phenylpropanoid metabolism was also conserved (green; Figure 6). In contrast, genes encoding cell wall proteins, fatty acid desaturation functions and oxidative pentose phosphate pathway proteins showed a conserved down-regulation response to bacterial, fungal and parasite infection (green; Figure 6). Overall, in response to bacterial infection, the earliest responses occur at the cell wall, where this significant down-regulation of transcripts encoding cell wall proteins and cellulose synthesis occurs, while a number of signalling proteins including kinases and G-proteins are up-regulated, as early as 24 HPI (Figure 3, Figure 6). Upon reception of these signals, NAC and WRKY transcription factors are up-regulated, along with genes known to be involved in the general biotic stress responses (Figure 6). The functional role for a number of these has been shown in response to biotic and abiotic stresses (Table 3). Therefore the genelists presented in this study represent genes that are highly likely to have a functional role in one or more biotic stress responses.

## Conclusions

To our knowledge, this is the first study in rice that has examined global transcriptomic responses to a variety of biotic and abiotic stresses in parallel to present a model for the biotic stress response(s) in rice and reveal signature genes that represent responses unique to each biotic stress, as well as identify novel genes, some even lineage specific, that are “universally” stress responsive. The small number of antagonistic responses between abiotic and biotic stresses observed in signalling and regulation components needs to be further investigated, as they may present a barrier to developing plants with resistance to abiotic and biotic stresses. It is now evidenced that specific signalling components, NAC and WRKY transcription factors, in addition to a number of other specific genes, represent common responses to biotic stresses. Furthermore, extensive data mining showed that many of the genes identified by the analyses in this study have been shown to have a functional role in stress resistance, given that knock-down/out or over-expression of these genes have previously been shown to result in altering stress tolerance of the resulting transgenic plants. Thus, the genes identified in this study provide precise targets to over-express or manipulate in order to develop multiple resistances to stress.

## Methods

### Rice growth and infections

The recurrent parent of the near-isogenic lines, IR24 and *Xa21* isogenic line, IRBB21 were chosen as the susceptible and resistance cultivars, respectively [76]. Seeds were sowed in the greenhouse at the China National Rice Research Institute (CNRRI). Philippine *Xoo* race 4 or PR4 (strain PXO71), which causes disease on IR24 (and not in IRBB21 [24]), was used to inoculate 35-day old plants. Fully expanded leaves in the main tiller from each plant were inoculated using the leaf clipping method (Kauffman et al., 1973). Leaf samples were collected from the infected leaves as well and two other leaves (Figure 1B).

### Microarray experiments

Total RNA was extracted using TRIzol® Reagent (Invitrogen, Carlsbad, CA, USA) according to the manufacturer’s instructions. The integrity of each RNA sample was examined by Agilent Lab-on-a-chip technology using the RNA 6000 Nano LabChip kit and a Bioanalyzer 2100 (Agilent Technologies, Santa Clara, CA, USA). Microarrays were carried out by the Shanghai Biotechnology Corporation using the One-Cycle Target Labeling kits and Affymetrix GeneChips, following manufacturers’ instructions. To validate the microarray data in this study, the expression levels of specific genes that have previously been shown to be induced in response to Xoo. infection [77-79] were compared to the response in this study. Additional file 1: Table S5 shows that the correlation between the induction seen in this study in comparison to previous studies. Notably, the study by Gan and colleagues [77] involved both microarray data and PCR validation of specific pathogenesis-related genes that were induced. It can be seen that these genes (LOC_Os01g28450.1, LOC_Os07g03730.1, LOC_Os12g36860.1) were also induced in this study, confirming the expected induction of these genes in response to Xoo. infection in rice.

### Microarray data analysis and public arrays

All CEL files were first normalized by MAS5 analysis to determine present/absent calls for each gene on each array. Only genes that were present in at least one time point (called present in =/>2 replicates) were then kept for further analysis. Partek Genomics Suite was used to normalise the data by GC-RMA, as carried out previously [80]. In order to statistically determine differential gene expression between the mock treated and infected samples, the GC-RMA normalized values were analysed using the Bayesian based, Cyber-T method [72,81]. Using Cyber-T, a statistical analysis of differential expression was carried out, where the GC-RMA normalised values were used as input for all the control and treated samples. In this way a p-value could be generated for each comparison and given that these tests were designed for high-throughput biological data, a method for dealing with false discovery rate was also incorporated in the form of a posterior probability of differential expression (PPDE). In this study, differential expression was defined where both p<0.05 and PPDE>0.96 (i.e. <5% false discovery rate), as done in previous studies [67,80]. Note that for the differential expression comparisons within the susceptible cultivar, the false discovery rate (FDR) cut-off prevented any significantly differentially expressed genes from meeting the stringent criteria that was consistently used for all other comparisons (p<0.05; PPDE>0.96). Thus, comparisons within the susceptible cultivar (Figure 2), was the only exception where differentially expressed genes were shown solely on the basis of p<0.05, and therefore should be viewed with caution. For all other comparisons, i.e. all those shown in Table 1, only significantly differentially expressed, including FDR corrected genes (i.e. that had both p<0.05 and PPDE>0.96) were included.

Note that in order to achieve comparability between the microarray data from the different sources, all the raw CEL files from this study as well as those downloaded from GEO (Table 1) were imported into Partek Genomics Suite (v6.5) and GC-RMA normalised in the same manner upon import to ensure numeric comparability across arrays. Intensity distributions and descriptive statistics were checked to confirm comparability between arrays. Thus, confirming that the raw data used for differential expression was comparable and free of outlier arrays. The GC-RMA normalised data was then used in the Cyber-T analysis for each comparison, where significant differential expression was determined in the same way as described above, i.e. p<0.05, PPDE>0.96.

Raw CEL files for the microarrays used in response to Xoo infection in this study have been submitted to GEO under the accession GSE43050.

### Gene annotations and Pageman analysis

The annotations of gene function were derived from the Rice Genome Annotation Project database, where putative functional descriptions can be found for all rice genes (http://rice.plantbiology.msu.edu/). For the Pageman functional annotation, a complex system of automated functional annotation and manual curating was involved in assigning function to the rice genes (details are described in [27]). Within Pageman, over-representation was determined using Fisher’s exact test, and z-scores are presented as colours, where a score of 1.96 represents a p-value of 0.05 [27].

### Motif analysis

The 1kb upstream DNA sequences for all genes in the rice genome were extracted from the MSU database (http://rice.plantbiology.msu.edu/). For each sub-set, the occurrence of all motifs shown in AGRIS and Athamap [41,42] were calculated for each subset, shown as a percentage, and compared to the percentage occurrence of each motif across all 1kb upstream sequences in the rice genome. Statistical significance of over-representation was determined using z-score analysis, as done previously [80].

## Abbreviations

ABA: Abscisic acid; ABRE: Abscisic acid response element; AP2/EREBP: Ethylene response element binding protein; bZIP: Basic leucine zipper; CAREs: *cis*-acting regulatory element(s); FDR: False discovery rate; HMI: Hours after mock infection; HPI: Hours post infection; HSE: Heat shock element; KD: Knock-down; KO: Knock-out; NAC: NAM (no apical meristem), ATAF (Arabidopsis Transciption Activation Factor), CUC (cup-shaped cotyledon); NB-LRR: nucleotide-binding site leucine-rich repeat; Os: *Oryza Sativa*; OsWAK: *Oryza Sativa* Wall Associated Kinase; Ox: Over-expressor; PPDE: Posterior probability of differential expression; PRR: Pathogen recognition receptors; ROS: Reactive oxygen species; R: Resistant/Resistance (gene); S: Susceptible; TCA: Tricarboxylic acid cycle; TF: Transcription factor; Xoo: *Xanthomonas oryzae* pv. *Oryzae*; Xoc: *Xanthomonas oryzae* pv. *Oryzicola*

## Competing interests

We have no competing interests.

## Authors’ contributions

RN conceived the project idea, carried out all the data analysis from raw files to the generation of figures and drafting the manuscript. J Whelan was involved in the writing of the manuscript. CW, JC, J Wu and HS were involved in carrying out the rice growth and *Xoo.* infections. All authors read and approved the final manuscript.

## Supplementary Material

Additional file 1 Contains all the differentially expressed genes in response to Xoo. infection, all information about the public arrays analysed in paralell, lists the core genes identified in Figure 3, and shows the validation of Xoo. infection. Click here for file
